# Lithium–nitrogen–hydrogen systems for ammonia synthesis: exploring a more efficient pathway using lithium nitride–hydride†

**DOI:** 10.1039/d2cc01345b

**Published:** 2022-04-25

**Authors:** Manoj Ravi, Joshua W. Makepeace

**Affiliations:** School of Chemistry, University of Birmingham Birmingham B15 2TT UK j.w.makepeace@bham.ac.uk

## Abstract

Ammonia synthesis chemistry with lithium–nitrogen–hydrogen materials is largely confined to pathways involving lithium hydride and lithium imide. Herein, we explore an alternate pathway featuring lithium nitride–hydride that shows more favorable characteristics from an activity, synthesis and cyclability perspective.

## Abstract 2

Lithium nitride–hydride offers advantages in stability, preparation method and activity for ammonia synthesis in a chemical looping regime.

## Graphical abstract

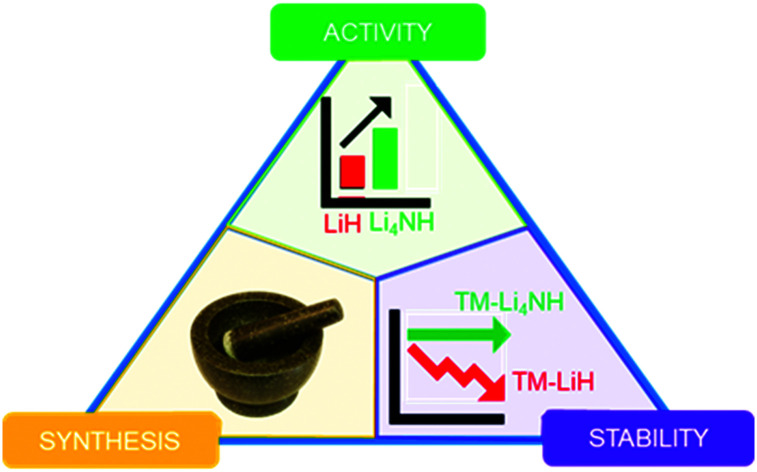

In the context of net zero emissions targets that need to be met to tackle climate change, the decarbonization of ammonia manufacture assumes significant importance. As a consequence of utilizing hydrogen derived from fossil fuel-feedstocks, the Haber-Bosch (HB) technology – the dominant industrial pathway for ammonia manufacture – contributes to nearly 2% of global greenhouse gas emissions.^1^ Besides decarbonizing the fertilizer industry, a greener pathway for ammonia synthesis will help unlock its use in diverse clean energy transition applications from transportation to grid-balancing.^2–5^ The high pressures and temperatures employed in the HB process make the technology more suited to centralized large-scale operation.^6,7^ In contrast, the economic viability and energy efficiency of green ammonia processes, which rely on intermittent renewable energy sources for green hydrogen, would benefit from the use of milder reaction conditions.^1,5,7^ However, conventional HB catalysts are not effective at lower temperatures and pressures,^8,9^ motivating the quest for better performing materials under these conditions.^10–12^

Among the many materials that have recently been investigated for this purpose, alkali and alkaline earth metal–nitrogen–hydrogen systems have shown considerable promise.^13–17^ When composited with a transition metal (TM), compounds such as lithium hydride (LiH) yield higher ammonia synthesis activity than HB catalysts under milder conditions.^11,18,19^ This is because these composites are more efficient at addressing the key challenge in ammonia synthesis, namely nitrogen activation. It has been proposed that they enable the ‘breaking’ of the scaling relationship between the N_2_ adsorption transition-state energy and the adsorption energy of NH_*X*_ intermediates (*x* = 0, 1 and 2) that governs the performance of conventional TM-based HB catalysts.^9,11^ In a TM-LiH composite, the hydridic hydrogen removes activated nitrogen from the TM, thereby serving as a second catalytic site that expedites nitrogen activation. When used in a chemical looping protocol, where nitrogen and hydrogen are fed sequentially, LiH is first converted to lithium imide (Li_2_NH), which on hydrogenation yields ammonia and regenerates lithium hydride. Hence, ammonia synthesis in this system is mediated by the interconversion of lithium hydride and lithium imide.^18^

Our objective is to exploit the chemical diversity of the Li-N-H family of materials to realize an alternative pathway for ammonia synthesis and discern the advantages associated with this route. This builds on our recent work where we highlighted the implications of compositional variation in the Li–N–H system for hydrogen storage and ammonia catalysis.^20^ More precisely, we revealed the formation of a solid solution with a highly disordered anti-fluorite structure for mixed lithium amide–imide (Li_2−*x*_NH_1+*x*_, 0 < *x* < 1) and lithium imide–nitride–hydride (Li_2+*x*_NH, 0 < *x* < 2) phases.^20^ While the former is more extensively studied from a hydrogen storage and release standpoint,^21^ the latter solid solution with nitride–hydride character is of interest to this study, considering the reported involvement of hydridic hydrogen in electron and hydrogen transfer during ammonia synthesis.^16^

Lithium nitride–hydride (Li_4_NH) was synthesized by heating a ball-milled mixture of lithium nitride (Li_3_N) and LiH in a microwave reactor (Experimental section, ESI†)^22^ and subsequently subject to a chemical looping ammonia synthesis process. On its own, Li_4_NH enables modest ammonia production rates, but at a level around 40% higher than LiH (1 bar gauge pressure, 400–500 °C, Fig. 1). We infer from the N_2_-Temperature-Programmed Reaction (N_2_-TPR) data that Li_4_NH fixes more nitrogen per unit mass of the material (Fig. 2 and Table 1) and per mol Li (Fig. S1, ESI†) than LiH, with the onset temperature of reactivity being much lower for Li_4_NH. While the idealized nitrogen fixing reactions in each of these materials point to the formation of Li_2_NH (eqn (1) and (2)), our experiments reveal significant nuance to the different chemical transformations that take place.

1 4LiH + N_2_ → 2Li_2_NH + 2H_2_

2 3Li_4_NH + N_2_ → 3Li_2_NH + 2Li_3_N

**Fig. 1:**
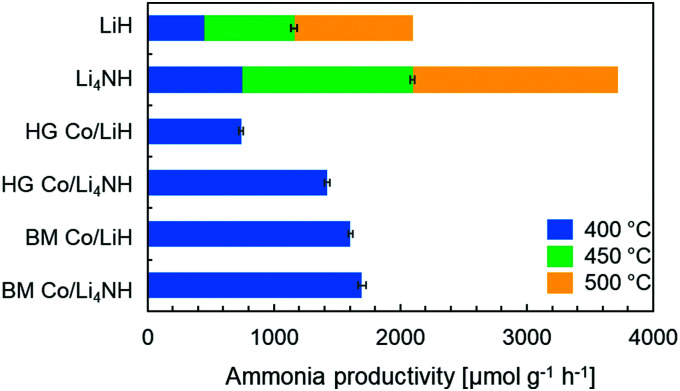
Ammonia synthesis rate with plain LiH, Li_4_NH and cobalt-containing samples in a chemical looping process. Reaction conditions: 60 sccm N_2_ followed by 75 sccm H_2_, 1 barg, 20 mg material. The stacked bars at 450 and 500 °C for LiH and Li_4_NH show the absolute rate and not the increase in rate from that observed at the lower temperature. HG = hand-ground, BM = ball-milled.

**Fig. 2:**
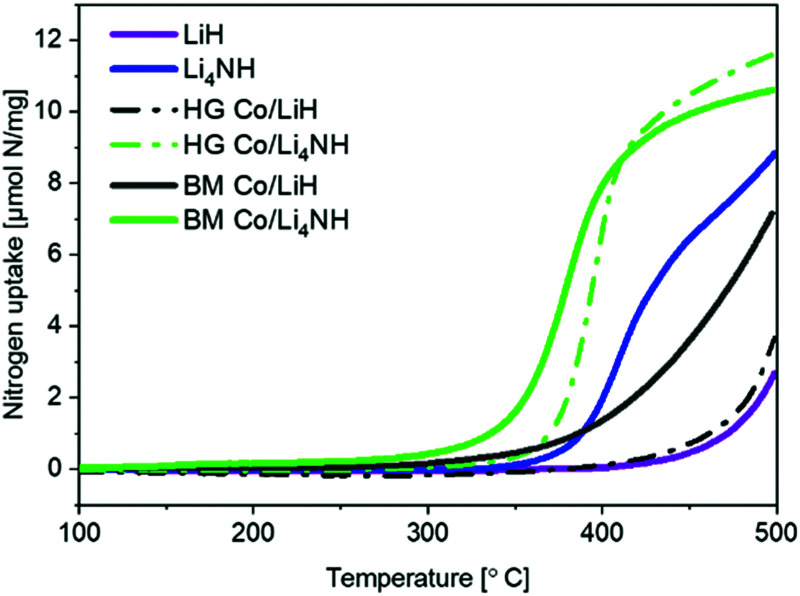
N_2_-TPR profiles of plain and cobalt-composited LiH and Li_4_NH. HG = hand-ground, BM = ball-milled.

**Table 1:** Nitrogen uptake and lattice parameter variation of *Fm*3̄*m* structure during nitrogen-fixing and hydrogenation steps. BM = ball-milled

Material	N-uptake^a^	*a* [Å] of *Fm*3̄*m* phase^b^ after		
	[μmol mg^−1^]	N_2_	N_2_–H_2_	N_2_–H_2_–N_2_
LiH	0.2	4.9744(4)	—	n.d.
Li_4_NH	6.7	5.023(1)	4.9726(4)	4.9861(6)
BM Co/LiH	3.3	4.976(1)	—	n.d.
BM Co/Li_4_NH	10.4	5.058(3)	4.983(2)	4.9907(5)

a Nitrogen uptake as measured in a TPR experiment (ramp to 400 °C at 5 °C min^−1^ followed by 20 min isothermal segment).

b Lattice parameters determined by Rietveld analysis of XRD data after reaction at 450 °C for LiH and Li_4_NH and 400 °C for cobalt-composited samples; n.d: not determined.

The reaction of Li_4_NH with nitrogen at 450 °C results in the formation of Li_3_N and a phase with an *Fm*3̄*m* structure (Fig. 3) having a lattice parameter value of 5.023(1) Å, with neither hydrogen nor ammonia being detected during the N_2_-TPR experiment (Fig. S2, ESI†). At a reaction temperature of 400 °C, the lattice parameter of this phase is 4.9849(4) Å. This suggests that fixing nitrogen in Li_4_NH does not yield a strictly stoichiometric Li_2_NH phase, which is characterized by an *Fd*3̄*m* structure at ambient temperature and a disordered *Fm*3̄*m* structure with a lattice parameter of 5.047 Å at elevated temperatures.^23–25^ Instead, a phase with an *Fm*3̄*m* structure and a varying lattice parameter value in the range of 4.93 to 5.03 Å is emblematic of a lithium imide–nitride–hydride solid solution, with a lattice parameter towards the higher end indicating an imide-rich solution.^20^ A small amount of the *Fm*3̄*m* phase is present in the as-synthesized Li_4_NH sample, but with a much lower lattice parameter of 4.9339(6) Å, representing very slightly off-stoichiometry lithium nitride–hydride.^20^ These assignments are corroborated by Raman spectroscopy. While the spectrum of the parent Li_4_NH has sharp features at low wavenumbers corresponding to phonon modes of the nitride–hydride phase, the same features are much broader and less well-defined for the sample after reaction under nitrogen (Fig. 4). Furthermore, the appearance of a broad feature at *ca.* 3150 cm^−1^ in the latter is a fingerprint for an imide N–H stretch (Fig. 4). These Raman features constitute the signature for a lithium imide–nitride–hydride solid solution, with the lattice parameter value revealing an imide-rich stoichiometry after the nitrogen-fixing step (*ca.* Li_2.3_NH).

**Fig. 3:**
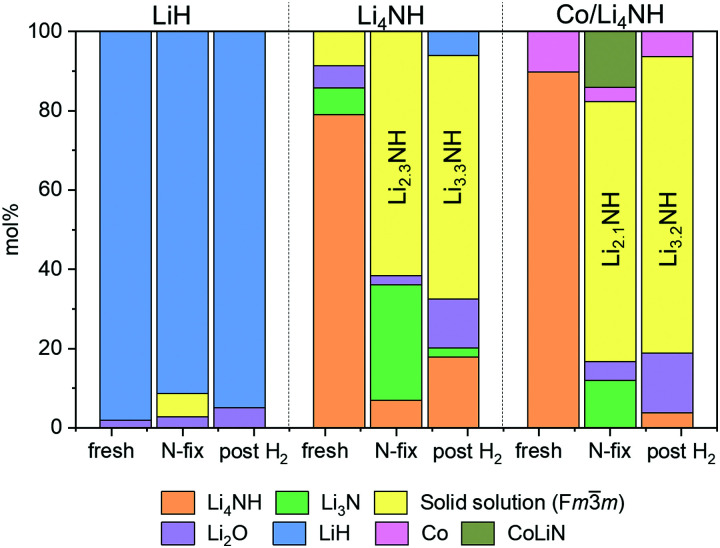
Phase composition as determined by Rietveld analysis of powder X-ray diffraction data. Raw and fit XRD data sets are provided in the ESI† (Fig. S4). Co and CoLiN phases are expected to be underestimates due to the use of a Cu X-ray source. Temperature of nitrogen-fixing and hydrogenation was 450 °C for LiH and Li_4_NH and 400 °C for Co/Li_4_NH. The composition of the *Fm*3̄*m* solid solution in the ‘N-fix’ and ‘post H_2_‘ samples is derived on the basis of the lattice parameter data (Table 1).^20^

**Fig. 4:**
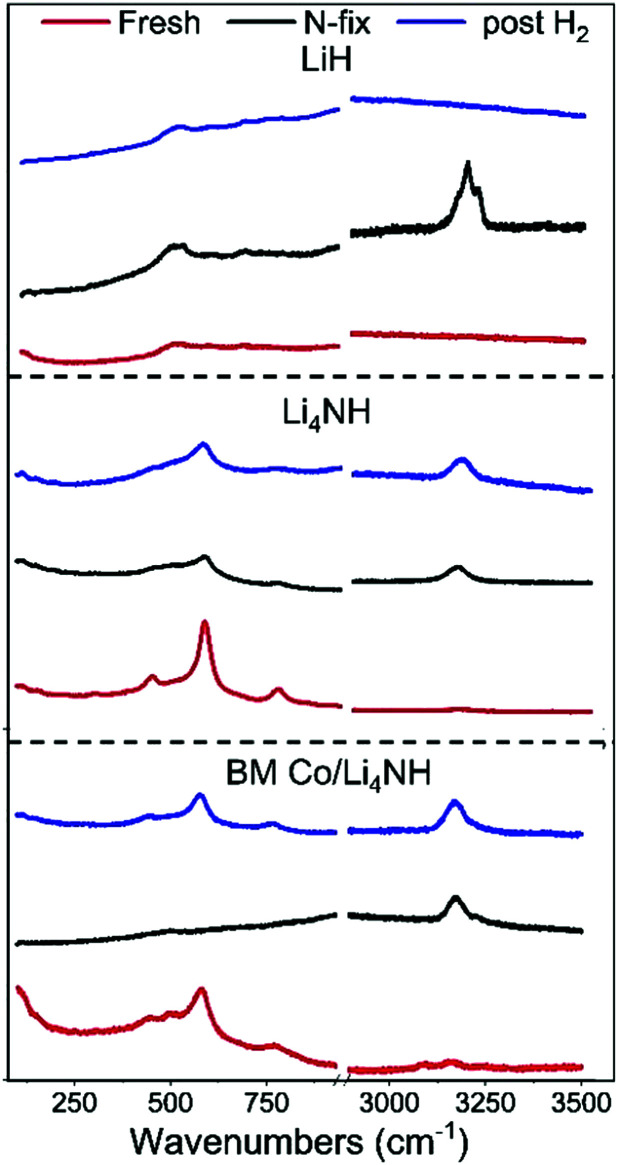
The low-energy phonon vibration region and the N–H stretching region of the Raman spectra of LiH, Li_4_NH and BM Co/Li_4_NH samples.

For LiH, reaction with N_2_ at 450 °C also results in a phase adopting an *Fm*3̄*m* structure (Fig. 3) but with a smaller lattice parameter than what was observed in the case of Li_4_NH (Table 1). Furthermore, the Raman spectrum for LiH after nitrogen-fixing shows a triplet feature in the N–H stretching region (Fig. 4), which appear to be consistent with reported data for a lithium imide-hydride mixed phase.^26^

During the hydrogenation phase, NH_x_ species formed during nitrogen fixing are converted into NH_3_. For LiH, this results in the disappearance of the *Fm*3̄*m* structure and regeneration of LiH (Fig. 3), with no features observed in the N–H stretching region of the Raman spectrum post reaction (Fig. 4).

In contrast, for the process starting with Li_4_NH, the hydrogenation step does not fully regenerate the starting material. Both the Raman spectra (Fig. 4) and XRD data (Fig. 3) reveal the continuing presence of the *Fm*3̄*m* solid solution. However, the lattice parameter of this phase decreases by approximately 0.05 Å on hydrogenation (Table 1), translating to a composition of *ca.* Li_3.3_NH for the solid solution. Hence, hydrogenation imparts a greater nitride–hydride character to the solid solution. However, prolonging the hydrogenation step does not convert the nitride–hydride-rich solid solution to pristine tetragonal Li_4_NH. Instead, it drives the undesired formation of LiH and a plateauing of the lithium imide-nitride–hydride solid solution lattice parameter at around 4.98 Å (Fig. S3, ESI†). This corresponds to the observed upper limit of nitride–hydride incorporation into the solid solution;^20^ further formation of stoichiometric Li_4_NH appears to result in rapid hydrogenation to LiH (Fig. S3, ESI†), which reduces the ammonia productivity in subsequent cycles. This highlights the need to abstain from forcing the solid solution into a composition regime where Li_4_NH isolates as a distinct tetragonal phase during hydrogenation, a condition which can be satisfied by judicious choice of cycle duration to sustain elevated productivity compared with LiH.

Next, we proceed to explore the effect of combining Li–N–H materials with a transition metal on the rate of ammonia synthesis. The composites were made either by hand-grinding the TM and LiH or Li_4_NH in a pestle and mortar (denoted by prefix HG) or ball-milling (denoted by prefix BM) the ingredients together (Experimental section, ESI†). Previous studies have shown that compositing LiH with a TM, such as cobalt, allows nitrogen to be fixed in the material at lower temperatures.^18^ Importantly, in LiH composites the synthesis route for introducing the TM is key to realizing better nitrogen uptake characteristics. While the composite prepared by hand-grinding (HG Co/LiH) shows minimal improvement compared to plain LiH, the more rigorous ball-milling route (BM Co/LiH) yields a much more favorable N_2_-TPR profile (Fig. 2). This difference in the nitrogen fixing behavior is borne out by the ammonia productivity data (Fig. 1). Hence, a good dispersion of the TM and small particle size are vital to unlock better performance, necessitating ball-milling over a simpler hand-grinding method.

In the case of Li_4_NH-based composites, the difference between the hand-ground and ball-milled samples both in the N_2_-TPR profiles (Fig. 2) and ammonia synthesis rate (Fig. 1) is much less stark than the LiH-based counterparts. Thus, a simpler hand-grinding method suffices to realize the promotion effect of the TM in the case of Co/Li_4_NH. This can be ascribed to the presence of nitrogen in the support that can anchor the TM and facilitate a more favorable metal-support interaction.^27^ As observed with plain Li_4_NH, the fixing of nitrogen in Co/Li_4_NH results in the formation of Li_3_N and the *Fm*3̄*m* phase (Fig. 3), which has a lattice parameter indicative of a composition very close to Li_2_NH, with significantly more nitrogen being fixed than in plain Li_4_NH at 400 °C. Furthermore, when fixing nitrogen in Co/Li_4_NH, we also observe the formation of lithium cobalt nitride (Co_0.333_Li_2.333_N), which disappears on subsequent hydrogenation (Fig. 3). The decrease in the lattice parameter of the *Fm*3̄*m* structure on hydrogenation is similar to that observed with Li_4_NH (Table 1), indicating the formation of a nitride–hydride-rich solid solution. Likewise, the Raman features are also very much comparable (Fig. 4).

As noted earlier, the hydrogenation step regenerates the starting material in LiH-based looping processes, which is not the case with Li_4_NH. While the hydrogenation of the lithium imide-rich solid solution back to Li_4_NH can be envisioned in theory, the susceptibility of tetragonal Li_4_NH to undergo further hydrogenation to LiH makes it challenging to experimentally realize an ideal looping process starting and finishing with Li_4_NH. Nevertheless, we saw that the compositional flexibility of the *Fm*3̄*m* solid solution, varying from Li_2_NH to *ca.* Li_3.3_NH, can be exploited to synthesize ammonia. The cycling within this compositional regime underpins the chemical looping process reported in this work (Fig. S5, ESI†). In the case of plain Li_4_NH as well as Co/Li_4_NH, the fixing of nitrogen in the material after one full cycle (N_2_–H_2_–N_2_ column, Table 1) results in a larger lattice parameter for the *Fm*3̄*m* phase relative to that observed after hydrogenation (N_2_–H_2_ column, Table 1), which signifies a return to greater imide character for the solid solution.

Previous studies using Ni–BaH_2_ composites have highlighted issues in cycling the material, with ammonia productivity falling by as much as 25% over 10 cycles.^18^ Our experiments with Co/LiH show similar issues in cyclability (Fig. 5). The impressive performance of these composites arises from the intimate contact between the TM and the hydride phase. The separation and aggregation of the TM and the hydride phase over multiple cycles is cited as the reason for the decreasing trend in ammonia productivity.^18^ In contrast to Co/LiH, the Co/Li_4_NH composites, both ball-milled and hand-ground, exhibit much more stable ammonia productivity over several cycles. This more favorable behavior is again likely to be driven by the presence of nitrogen in the support.^27^ As stated earlier, the ability of nitrogen to anchor the transition metal and facilitate metal-support interaction is expected to deter the separation and aggregation of the two phases. The X-ray diffraction patterns of the spent Co/Li_4_NH after 10 cycles show no features for LiH formation, which demonstrates the successful cycling of the material within the compositional regime of the imide-nitride–hydride solid solution for stable ammonia production. (Fig. S6, ESI†)

**Fig. 5:**
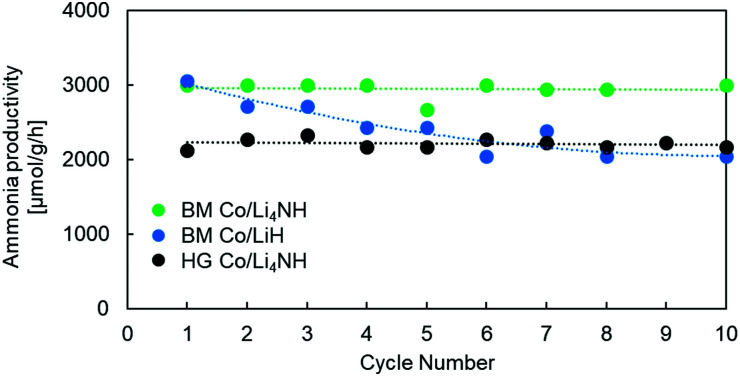
Cyclability tests of Co/LiH and Co/Li_4_NH samples. Reaction conditions for one cycle: 60 sccm N_2_ followed by 75 sccm H_2_, 1 barg, 450 °C, 15 mg material. HG = hand-ground, BM = ball-milled.

In summary, we have demonstrated a novel pathway for ammonia synthesis starting from lithium nitride–hydride. The looping process for ammonia synthesis was shown to be mediated by the solid solution formed between lithium nitride–hydride and lithium imide. This route produced greater rates of ammonia production than with lithium hydride, which appears to be related to a higher intrinsic nitrogen fixing capability Furthermore, when used in composite with a transition metal to enhance productivity, lithium nitride–hydride appears to more readily yield a well-dispersed material that is characterized by a more stable performance when tested for ammonia synthesis over multiple cycles.

The authors acknowledge the MRC for funding through a UKRI Future Leaders Fellowship (MR/S03403X/1). Dr Tzu-Yu Chen and Dr Louise Male are thanked for technical support. MR thanks Jeremy Lowen for help with the synthesis of lithium nitride-hydride. Raw data used in this work are available *via* UBIRA (https://doi.org/10.25500/edata.bham.00000827).

## Conflicts of interest

There are no conflicts to declare.

## Supplementary Material

CC-058-D2CC01345B-s001
