# Supplementary material for: Lithium–nitrogen–hydrogen systems for ammonia synthesis: exploring a more efficient pathway using lithium nitride–hydride
Source: Chem Commun (Camb). 2022 Apr 25;58(41):6076–9. doi: 10.1039/d2cc01345b (PMC9121210; doi:10.1039/d2cc01345b)
Supplement: CC-058-D2CC01345B-s001 [file CC-058-D2CC01345B-s001.pdf]

Electronic Supplementary Material (ESI) for ChemComm.
This journal is © The Royal Society of Chemistry 2022

Electronic Supplementary Information

Lithium-nitrogen-hydrogen systems for ammonia synthesis: exploring a more efficient pathway using lithium nitride-hydride

Manoj Ravi and Joshua W. Makepeace

School of Chemistry, University of Birmingham, B15 2TT, UK

Experimental Section

Synthesis of lithium nitride-hydride

Lithium nitride-hydride was synthesized by adopting the protocol developed by Tapia-Ruiz et al.[1] Firstly, lithium nitride (Merck, >99.5%) and lithium hydride (Merck, 95%) taken in appropriate stoichiometric amounts (0.8142 g of lithium nitride and 0.1858 g of lithium hydride for 1 g of lithium nitride-hydride) are hand-ground in a pestle and mortar for 5 minutes. The hand-ground mixture is then transferred into a 45 mL silicon nitride grinding jar containing 20 g of 5 mm diameter tungsten carbide balls for 1 g of the reactants. The milling was carried out at 400 rpm for 1 hour, performed as 12 cycles of 5 minutes each with a 2-minute pause between cycles (Fritsch Pulverisette 7 Premium Line). The recovered mixture is then pelletized to yield pellets of ca. 0.2 g. Such pellets are then transferred to a microwave reactor (CEM Discover SP) and subjected to five 1-minute cycles at a power of 300W. The temperature during reaction typically goes up to about 393 K. The pellets post reaction are crushed to yield lithium nitride-hydride in the powder form.

Synthesis of transition metal-lithium hydride and lithium nitride-hydride composites

The composites of transition metal (typically, cobalt) with lithium hydride and lithium nitride-hydride were made by two ways: hand-grinding and ball-milling. In the hand-grinding method, appropriate amounts of the transition metal and support material, for example, 0.2 g of cobalt powder (Alfa Aesar, 1.6-micron, 99.8% metals basis) and 0.8 g of lithium hydride for a 20 wt% Co/LiH composite, are ground together by hand in a pestle and mortar for 20 minutes. Such composites are denoted using the prefix HG. In the ball-milling method, mixtures of the transition metal and lithium hydride or lithium nitride-hydride are ball-milled at 400 rpm typically for 3 hours (36 cycles of 5 minutes each with a 2-minute pause between cycles and using 20 g of mill balls per g of solid to be milled). Such composites are denoted using the prefix BM.

Chemical looping experiments

The chemical looping process for ammonia production was carried out as follows: The material of interest is loaded in a stainless-steel tube-in-tube reactor (Cambridge Reactor Designs) inside an argon atmosphere glove box. A schematic of the reactor is shown below. The reactor is then attached to a rig and heated to the desired reaction temperature under nitrogen flow (BOC Nitrogen research grade N5.5). After exposure to nitrogen flow for a fixed period of time, the gas flow is switched to hydrogen (BOC Hydrogen research grade N5.5). During the entire experiment, a back-pressure regulator is used to control the operating pressure. The gas stream leaving the reactor is passed into an acid trap containing diluted sulphuric acid (2.5 mM, 500 mL) solution whose ionic conductivity was monitored online using a conductivity meter (Thermo Fisher Orion Star). As reported previously,[2] the decrease in the ionic conductivity is used to compute the ammonia productivity based on a calibration developed for change in conductivity with the amount of ammonia bubbled into the acid solution.

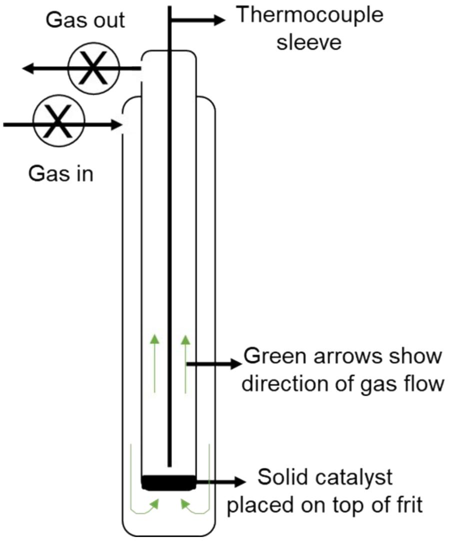

Temperature programmed reaction

Nitrogen uptake temperature programmed reactions were performed on the Netzsch 449F1 Jupiter thermogravimetric instrument. Around 20 mg of sample is added to an alumina crucible in an argon atmosphere glove box, covered with an alumina lid with a vent hole and placed in a vial before taking it to the instrument for analysis. The crucible is transferred to the instrument as quickly as possible to prevent exposure to ambient conditions. Soon thereafter, the gas atmosphere within the furnace containing the crucible is evacuated and refilled with nitrogen for a total of 3 cycles. The sample is then heated under flowing nitrogen (50 mL/min) to 773 K at a rate of 5 K/min with the mass being recorded online at a frequency of 4 points/min.

X-ray diffraction

Powder X-ray diffraction data were collected using the Siemens D5000 powder diffractometer with a monochromated Cu-Kα_1 X-ray source. Polyimide tubing (Cole-Parmer), with an inside diameter of 0.0340(5)" and an outside diameter of 0.0380(5)", sealed with superglue on one end and vacuum grease on the other, was used to hold the sample for XRD analysis. All XRD data were analysed by Rietveld analysis using the TOPAS Academic package (v6).

Raman spectroscopy

Spectroscopic analysis of the samples was performed using a Raman microscope (Renishaw inVia). Each sample was loaded into a 0.7 mm wide borosilicate glass capillary to a height of approximately 10 mm and sealed with grease. Each capillary was then individually loaded onto the adjustable table, moved into position and brought into focus using the built-in microscope with x20 lens. Raman data were collected using a 532 nm laser and a suitable aperture to ensure sampling across multiple powder grains.

Figure S1. N_2-TPR profiles of plain and cobalt-composited LiH and Li_4NH showing nitrogen uptake per mol Li.

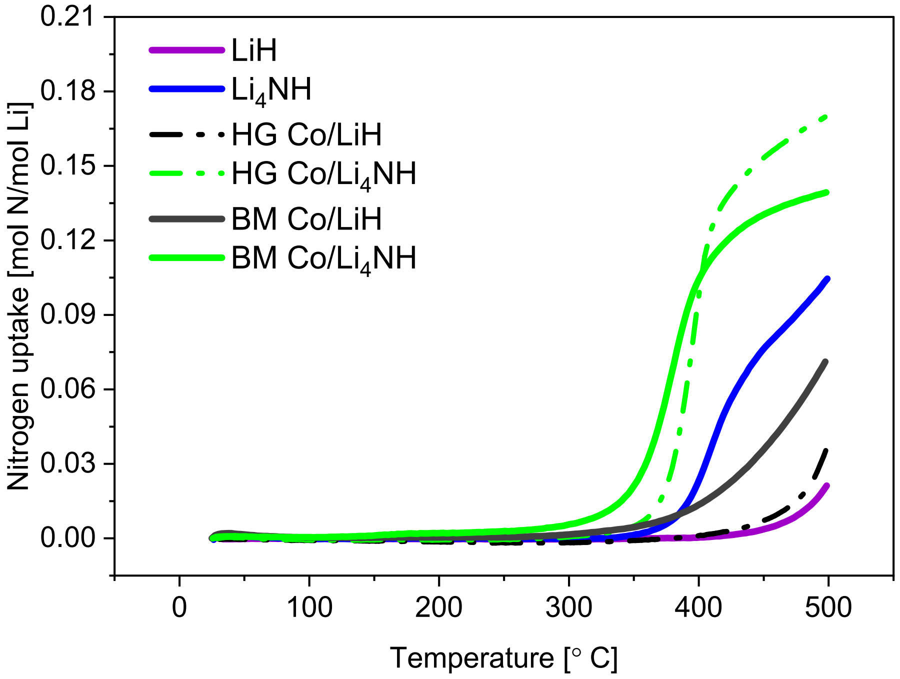

The trends in the N_2-TPR profiles in terms of mol N/mol Li are comparable to nitrogen uptake per unit mass of the material shown in Fig. 2.

Figure S2. Mass spectrometry signals for hydrogen and ammonia during the N_2-TPR experiment of lithium nitride-hydride

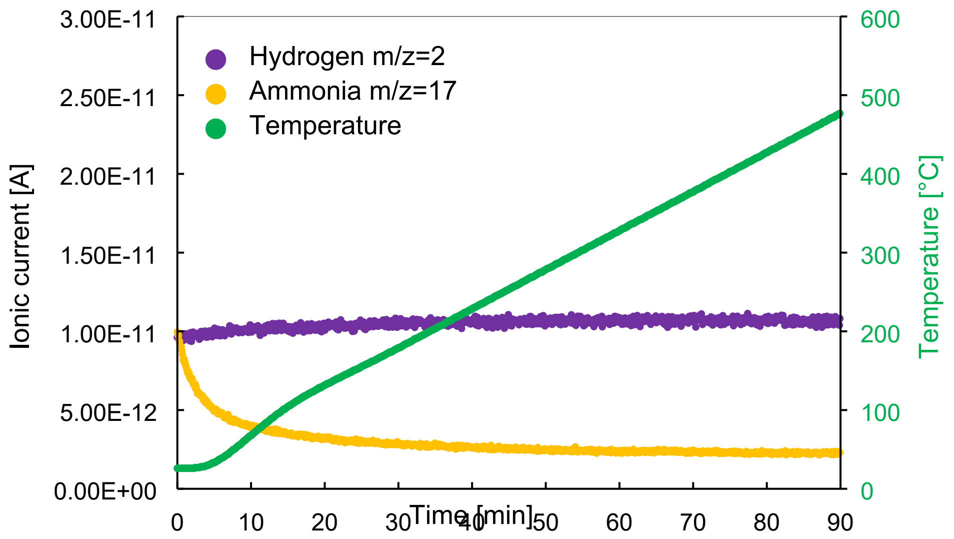

The MS signal traces for m/z ratio of 2 and 17, representing hydrogen and ammonia respectively, show the absence of evolution of these gaseous products during nitrogen-fixing in lithium nitride-hydride.

Figure S3. Effect of hydrogenation time on the formation of lithium hydride in the chemical looping of lithium nitride-hydride

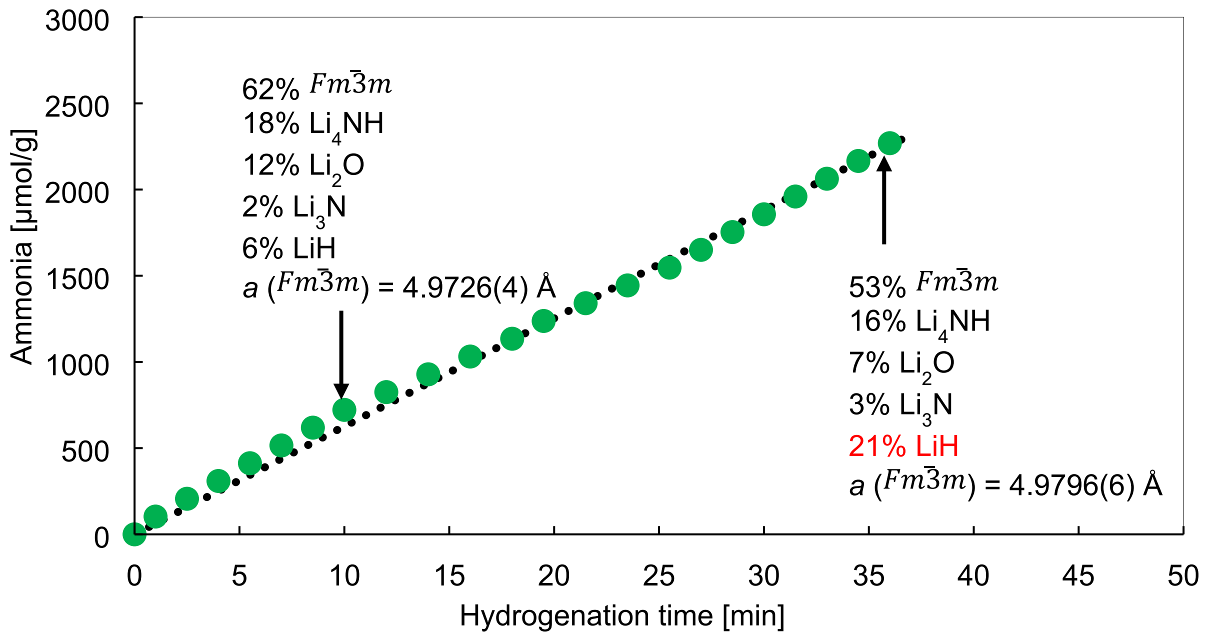

Subjecting the sample to hydrogenation for long times does not increase the amount of lithium nitride-hydride but results in the increased formation of undesired lithium hydride. The sample composition shown in mol% at the two points are obtained from the Rietveld analysis of the corresponding X-ray diffractograms. The lattice parameter value of the $Fm\bar{3}m$ structure corresponds to the observed upper limit of nitride-hydride incorporation in the solid solution.[3] For such and more nitride-hydride-rich compositions, tetragonal lithium nitride-hydride is formed as a distinct phase in addition to the $Fm\bar{3}m$ structure, and this lithium nitride-hydride appears to undergo hydrogenation to form lithium hydride.

Figure S4. Fitted XRD patterns

In all of the following figures, collected data are shown in black, fit by Rietveld analysis in red, and difference in blue.

The composition of the samples in terms of mol% obtained from the Rietveld analysis are shown in Fig. 3

Tick mark colours are as follows: Tetragonal Li_4NH = green, $Fm\bar{3}m$ solid solution = black, α-Li_3N = orange, Li_2O = cyan, LiH = magenta, cobalt fcc = red, cobalt hcp = brown, lithium cobalt nitride = olive

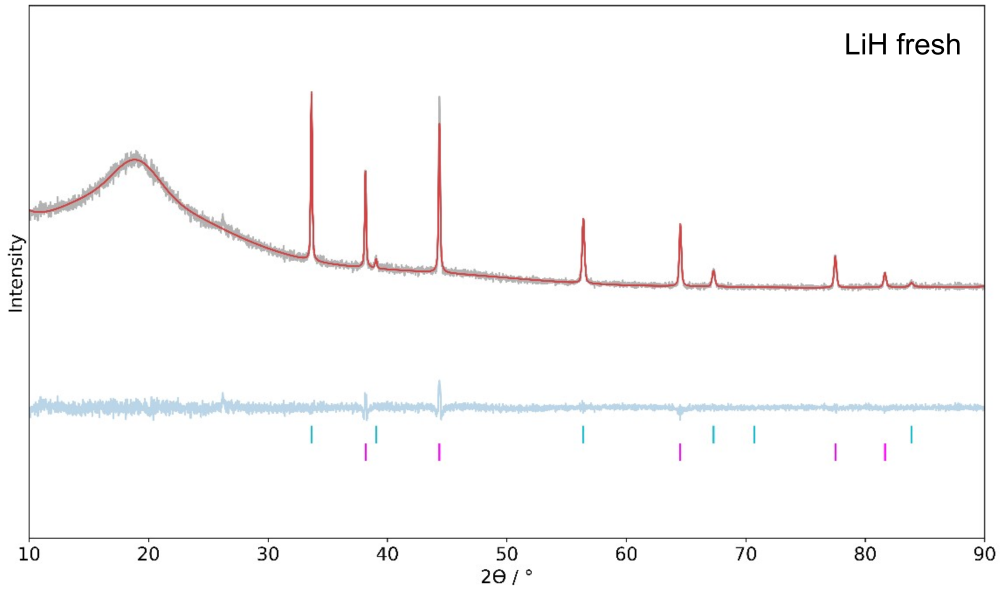

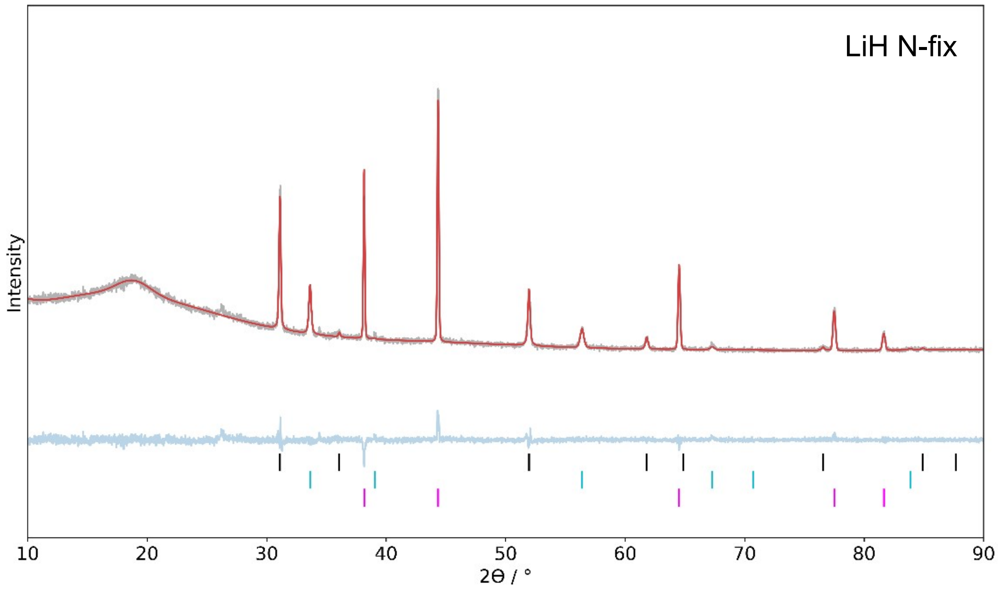

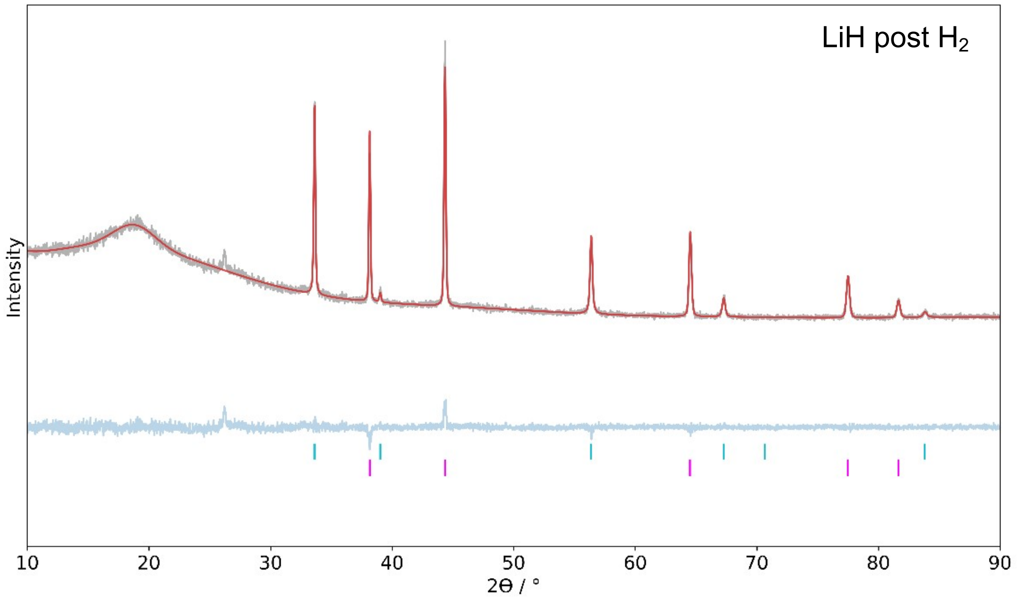
LiH post H_2
Intensity
10
20
30
40
50
60
70
80
90
2Θ / °

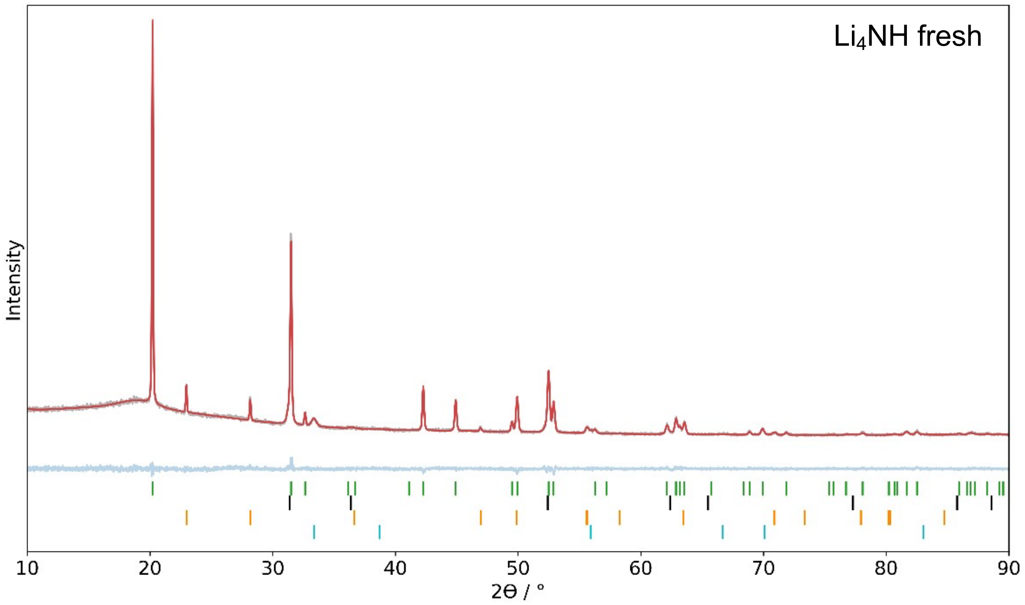
Li_4NH fresh
Intensity
10
20
30
40
50
60
70
80
90
2Θ / °

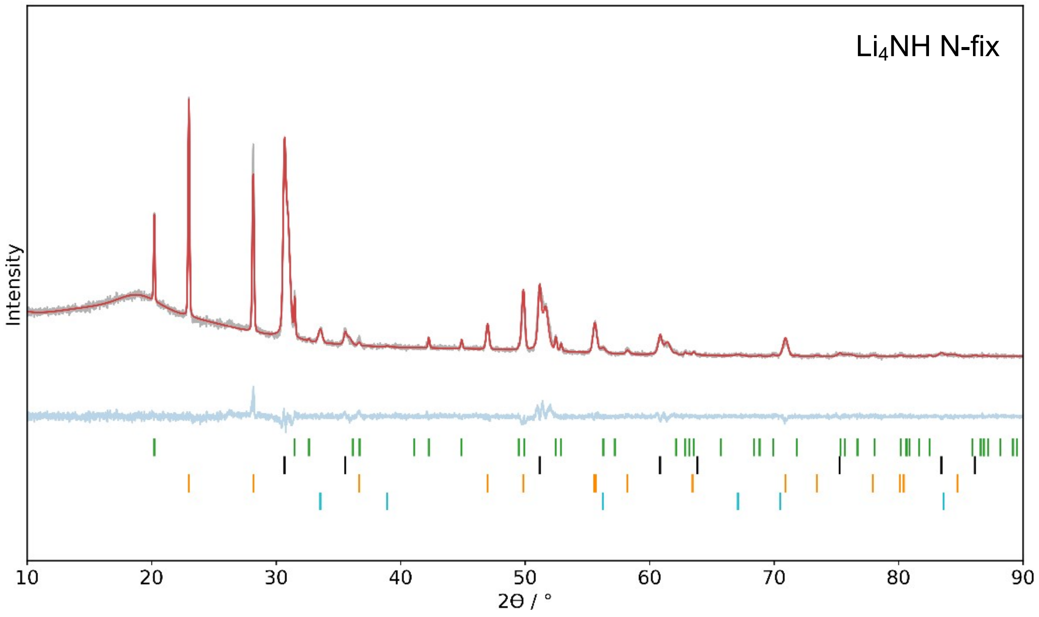
Li_4NH N-fix
Intensity
10
20
30
40
50
60
70
80
90
2Θ / °

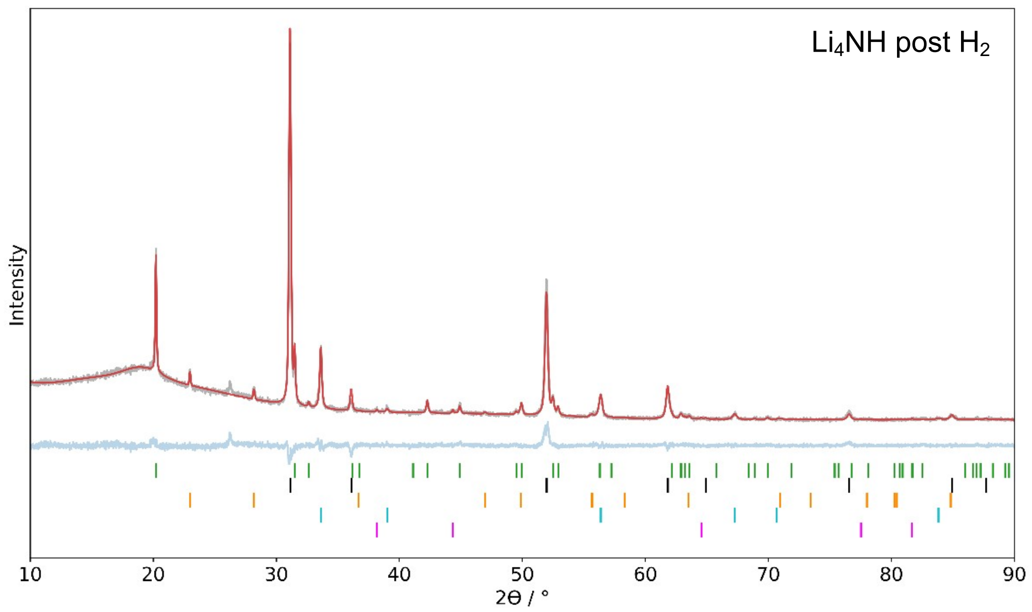

Li_4NH post H_2
Intensity
10
20
30
40
50
60
70
80
90
2Θ / °

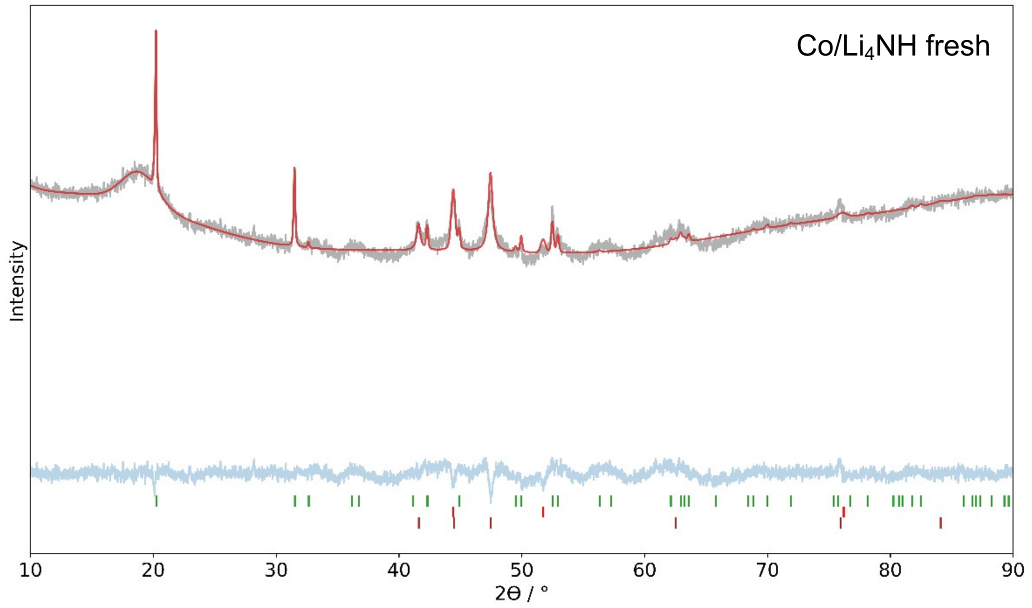

Co/Li_4NH fresh
Intensity
10
20
30
40
50
60
70
80
90
2Θ / °

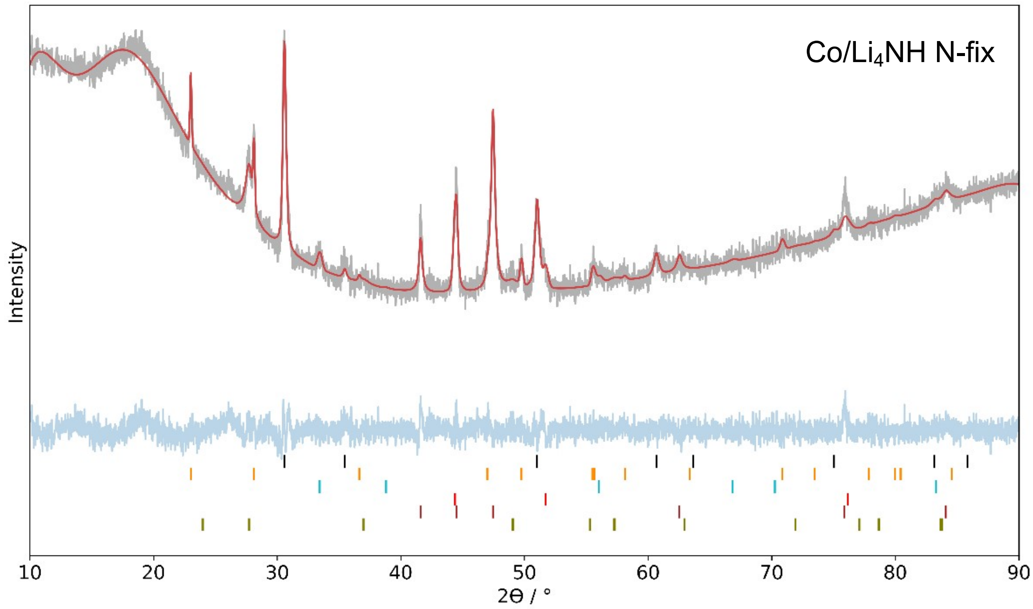
Co/Li4NH N-fix
Intensity
10
20
30
40
50
60
70
80
90
2Θ / °

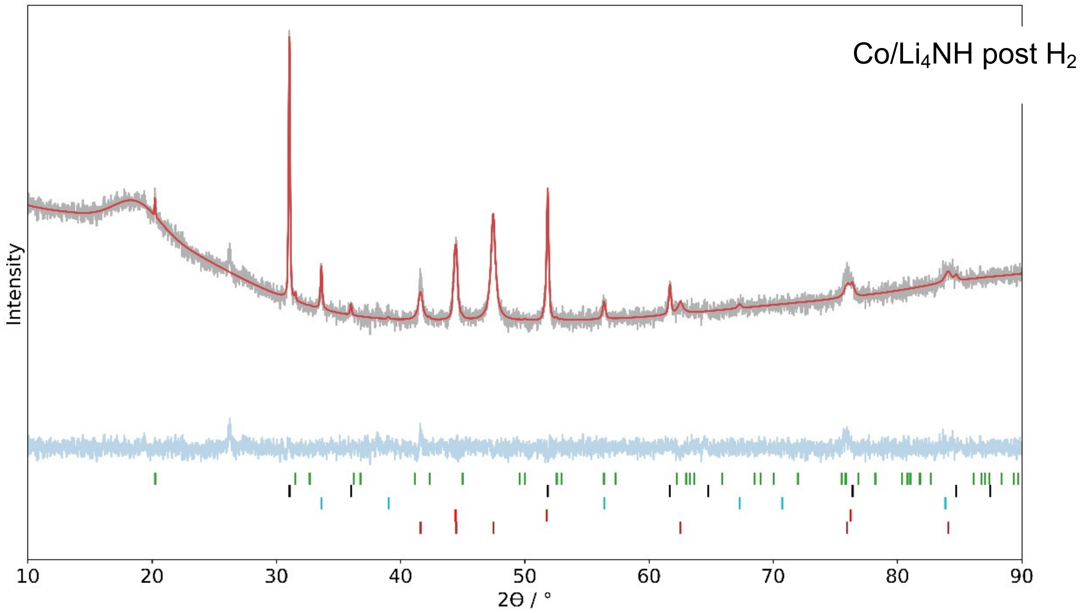
Co/Li4NH post H2
Intensity
10
20
30
40
50
60
70
80
90
2Θ / °

Figure S5. Reaction scheme for chemical looping based on lithium nitride-hydride

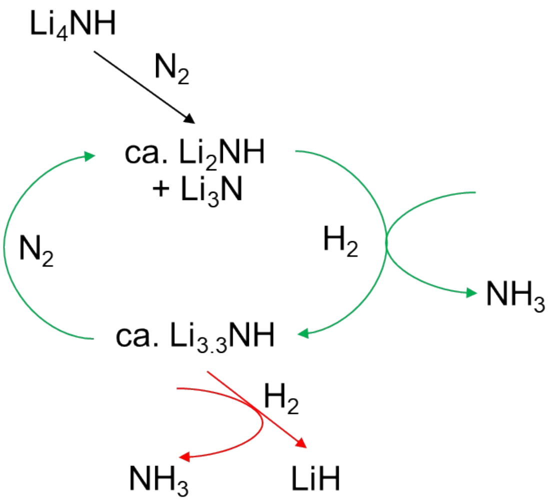

The reaction scheme is developed on the basis of the experimental data presented in the manuscript. In this system, the chemical looping process for ammonia production exploits the possibility to cycle between the imide-rich and nitride-hydride-rich compositions of the lithium imide-nitride-hydride solid solution.

Figure S6. X-ray diffraction data of spent Co/Li_4NH after 10 cycles

Collected data is shown in black, fit by Rietveld analysis in red, and difference in blue. Tick mark colours are as follows: Tetragonal Li_4NH = green, $Fm\bar{3}m$ solid solution = black, Li_2O = cyan, cobalt fcc = red and cobalt hcp = brown

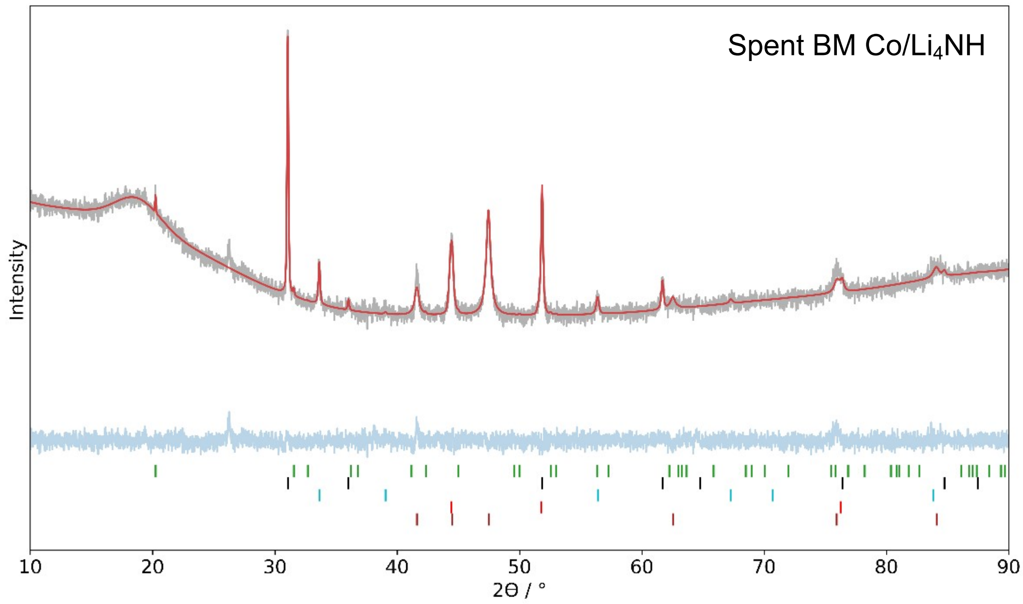

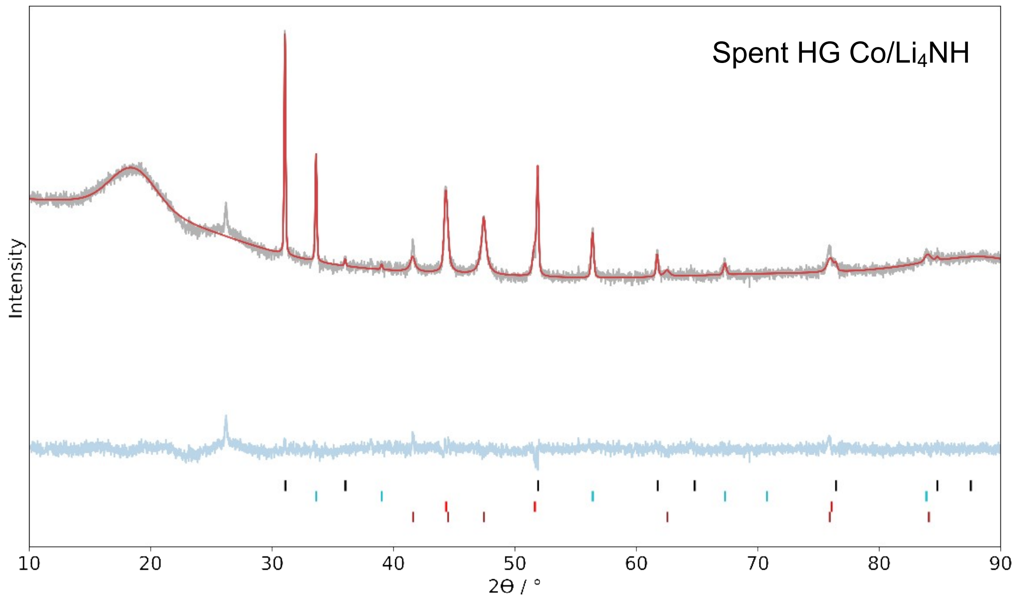

The X-ray diffraction patterns of the spent BM and HG Co/Li_4NH after 10 cycles show features for cobalt, the $Fm\bar{3}m$ solid solution, lithium nitride-hydride and lithium oxide. No diffraction peaks corresponding to lithium hydride are observed. Therefore, the cyclability in ammonia production arises from looping the material within the compositional flexibility offered by the solid solution between the lithium imide and lithium nitride-hydride.

References

1. N. Tapia-Ruiz, N. Sorbie, N. Vaché, T. K. A. Hoang and D. H. Gregory, *Materials*, 2013, **6**, 5410-5426.
2. W. Gao, J. Guo, P. Wang, Q. Wang, F. Chang, Q. Pei, W. Zhang, L. Liu and P. Chen, *Nature Energy*, 2018, **3**, 1067-1075.
3. J. W. Makepeace, J. M. Brittain, A. S. Manghnani, C. A. Murray, T. J. Wood and W. I. F. David, *Physical Chemistry Chemical Physics*, 2021, **23**, 15091-15100.
